# Effect of Polyunsaturated Fatty Acids and Their Metabolites on Bleomycin-Induced Cytotoxic Action on Human Neuroblastoma Cells *In Vitro*

**DOI:** 10.1371/journal.pone.0114766

**Published:** 2014-12-23

**Authors:** Sailaja Polavarapu, Arul M. Mani, Naveen K. V. Gundala, Anasuya D. Hari, Siresha Bathina, Undurti N. Das

**Affiliations:** 1 Bio-Science Research Centre, Gayatri Vidya Parishad College of Engineering Campus, Madhurawada, Visakhapatnam-530048, Andhra Pradesh, India; 2 UND Life Sciences, 2020 S 360^th^ St, # K-202, Federal Way, Washington, 98003, United States of America; Southern Illinois University School of Medicine, United States of America

## Abstract

In the present study, we noted that bleomycin induced growth inhibitory action was augmented by all the polyunsaturated fatty acids (PUFAs) tested on human neuroblastoma IMR-32 (0.5×10^4^ cells/100 µl of IMR) cells (EPA> DHA> ALA = GLA = AA> DGLA = LA: ∼60, 40, 30, 10–20% respectively) at the maximum doses used. Of all the prostaglandins (PGE_1_, PGE_2_, PGF_2α_, and PGI2) and leukotrienes (LTD_4_ and LTE_4_) tested; PGE_1_, PGE_2_ and LTD_4_ inhibited the growth of IMR-32 cells to a significant degree at the highest doses used. Lipoxin A_4_ (LXA_4_), 19,20-dihydroxydocosapentaenoate (19, 20 DiHDPA) and 10(S),17(S)-dihydroxy-4Z,7Z,11E,13Z,15E,19Z-docosahexaenoic acid (protectin: 10(S),17(S)DiHDoHE), metabolites of DHA, significantly inhibited the growth of IMR-32 cells. Pre-treatment with AA, GLA, DGLA and EPA and simultaneous treatment with all PUFAs used in the study augmented growth inhibitory action of bleomycin. Surprisingly, both indomethacin and nordihydroguaiaretic acid (NDGA) at 60 and 20 µg/ml respectively enhanced the growth of IMR-32 cells even in the presence of bleomycin. AA enhanced oxidant stress in IMR-32 cells as evidenced by an increase in lipid peroxides, superoxide dismutase levels and glutathione peroxidase activity. These results suggest that PUFAs suppress growth of human neuroblastoma cells, augment growth inhibitory action of bleomycin by enhancing formation of lipid peroxides and altering the status of anti-oxidants and, in all probability, increase the formation of lipoxins, resolvins and protectins from their respective precursors that possess growth inhibitory actions.

## Introduction

Previously, we and others showed that several polyunsaturated fatty acids (PUFAs) have selective cytotoxic action on many tumor cells of different types with little or no action on normal cells [1]–[14]. But, PUFAs themselves are not very effective in eliminating cancer cells in an *in vivo* situation partly, due to the fact that they are tightly bound to albumin and other proteins and hence, are unavailable to bring about their tumoricidal action [15]–[17]. Furthermore, PUFAs may be metabolized into several eicosanoids that may have other unwanted actions. Hence, it is desirable to develop methods whereby PUFAs are selectively delivered to tumor cells to produce their anti-cancer actions and/or given in combination with anti-cancer drugs so that the combined anti-cancer drug(s)+PUFAs may have a significant cytotoxic action on cancer cells compared to either agent alone. Studies showed that indeed a combination of PUFAs and conventional anti-cancer drugs have more potent action on tumor cells compared to either compound alone [18]–[23]. Some studies suggested that the tumoricidal action of PUFAs is not dependent on the formation of cyclo-oxygenase (COX) and lipoxygenase (LOX) products though, this has been disputed [1], [2], [24]–[28]. This uncertainty of the involvement of COX and LOX products on the growth/apoptosis of tumor cells is further supported by the observation that different prostaglandins either enhance or inhibit growth depending on the dose and type of the compounds tested and much less is known about the action of leukotrienes and thromboxanes on cancer cells [29]–[42]. In this context, it is noteworthy that effect of lipoxins derived from AA; resolvins from EPA and DHA and protectins from DHA on the growth of tumor cells has not been well evaluated though some studies did suggest that they may have anti-proliferative properties [43]–[47]. Many of these studies did not evaluate direct action of prostaglandins, leukotrienes, lipoxins, resolvins and protectins on the growth of tumor cells and much less is known about the effect of pre- and simultaneous treatment of tumor cells with PUFAs and their eicosanoid products on the anti-proliferative action of conventional anti-cancer drugs. In the present study, we evaluated the effect of various PUFAs, prostaglandins, leukotrienes, lipoxins, resolvins and protectins on the proliferation of human neuroblastoma (IMR-32) cells *in vitro* and compared these results to those obtained with COX and LOX inhibitors. The modulatory influence of PUFAs, prostaglandins, leukotrienes, lipoxins, resolvins and protectins on bleomycin-induced growth inhibitory action on IMR-32 cells was also studied. Finally, we evaluated the effect of AA, as a representative of unsaturated lipids, and bleomycin on anti-oxidant content, formation of lipid peroxides and nitric oxide in IMR-32 cells.

## Materials and Methods

### Reagents

All culture media and additives were purchased from Sigma Aldrich Chemicals Pvt. Ltd., Bangalore, India. Bleomycin was purchased from Cipla, Goa, India. All PUFAs and their metabolites (Prostaglandins, Leukotrienes, Lipoxin A4, Protectins and Resolvins) used in the present study were purchased from Cayman Chemical Company, Michigan, USA.

### Cell culture conditions

Human neuroblastoma cell line (IMR-32) obtained from Center for Cellular and Molecular Biology, Hyderabad, India (origin of source, ATCC) was grown in DMEM (pH 7.4) supplemented with bicarbonate, 100 U/ml penicillin, 100 µg/ml streptomycin, 1.25 µg/ml amphotericin B, 10% FBS at 37°C with 5% CO_2_.

IMR-32 grows as a monolayer and was subcultured when they became confluent. For culture experiments, cells were harvested from the confluent flask by washing them with phosphate buffered saline (PBS, pH 7.4) and treating with Trypsin (0.25%) – EDTA (0.02%) for 3 minutes. Trypsin was immediately inactivated by addition of equal volume of FBS and centrifuged to pellet the cells which were used for various studies as described below.

### Effect of bleomycin on the proliferation of IMR-32 cells *in vitro*

IMR-32 cells were plated at a density of 0.5 × 10^4^ cells/100 µl of culture media in 96-well plates. After 48 hrs of attachment period, cells were treated with different doses of bleomycin (7.5–120 µg/ml) for different periods of time (12–48 Hrs). At the end of each treatment period, viable cell numbers were measured by MTT (3-(4,5-dimethylthiazole-2-yl)-2,5-diphenyltetrazolium bromide) assay. Briefly, after the treatment, spent media was carefully removed and 100 µl of MTT (0.5 mg/ml) was added to each well and incubated for 4 hrs at 37°C with 5% CO_2_. Then, 100 µl of 10% acidified sodium dodecyl sulfate (SDS) solution was added to each well and incubated for further 18 hrs to dissolve the formazan crystals produced. The dissolved formazan product was measured as absorbance at A570 nm and the background at A620 nm using a 96-well ELISA plate reader (Multiskan EX, Thermo Scientific Ltd, USA). The cell growth percentage was expressed as the percentage of cell growth compared with control in the same treatment group.

### PUFAs and their metabolites on the proliferation of IMR-32 cells *in vitro*

IMR-32 cells were plated at a density of 0.5 × 10^4^ cells/100 µl of culture media in 96-well plates. After 48 hrs of attachment period, cells were treated with different doses of various PUFAs and their metabolites for 24 hours. At the end of the incubation period, the viable cells were measured by MTT assay as detailed above.

### Effect of PUFAs and their metabolites on bleomycin induced cytotoxicity to IMR-32 Cells

Two types of experiments were carried out to study the effects of various PUFAs/lipoxinA4/resolvins/protectins/prostaglandins/leukotrienes on bleomycin induced cytotoxicity as described below. The concentration of bleomycin 60 µg/ml, which produced ∼50% cytotoxicity to IMR-32 cells at the end of 24 hours of incubation, was selected for various experiments.

### Pre-treatment studies

After 48 hrs of initial attachment period, cells seeded in 96-well plate and were first incubated with different doses of PUFAs (10, 20, 30 µg/ml)/lipoxin A4 (1, 5, 10 ng/ml)/resolvins (1, 5, 10 ng/ml)/protectins (1, 5, 10 ng/ml)/prostaglandins (10, 50, 100 ng/ml)/leukotrienes (10, 50, 100 ng/ml) for 5 hrs. After 5 hrs, spent media was replaced with fresh media containing bleomycin (60 µg/ml) and IMR-32 cells were incubated for an additional 24 hrs. At the end of the treatment period, viable cell numbers were measured by MTT assay as described above.

### Simultaneous treatment

After 48 hrs of initial attachment period, IMR-32 cells seeded in 96-well plate as described above with plain culture media for 5 hrs. After 5 hrs, cells were treated simultaneously with different doses of PUFAs (10, 20, 30 µg/ml)/lipoxin A4 (1, 5, 10 ng/ml)/resolvins (1, 5, 10 ng/ml)/protectins (1, 5, 10 ng/ml)/prostaglandins (10, 50, 100 ng/ml)/leukotrienes (10, 50, 100 ng/ml) and bleomycin (60 µg/ml) and incubated for an additional 24 hrs. At the end of the treatment period, viable cell numbers were measured by MTT assay.

### Effect of cyclo-oxygenase (COX) and lipoxygenase (LOX) inhibitors on arachidonic acid (AA) mediated effect on bleomycin induced cytotoxicity to IMR-32 Cells

After 48 hrs of initial attachment period, IMR-32 cells were seeded in 96-well plate as described above and treated simultaneously with bleomycin and AA in the presence of different doses of indomethacin (COX inhibitor; 20, 40 and 60 µg/ml) or nordihydroguaiaretic acid (NDGA, LOX inhibitor; 5, 10 and 20 µg/ml) and incubated for 24 hours. At the end of the treatment period, viable cells were measured by MTT assay as described above.

### Assay of antioxidant enzymes, lipid peroxides, nitric oxide (NO)

IMR-32 cells were plated at a density of 5 × 10^4^ cells/ml of culture media in 24-well plates. After 48 hours of attachment period, cells were treated with bleomycin and AA for an additional 24 Hrs. At the end of the treatment period, supernatant was collected and cells were washed with PBS (pH 7.4). Cells were lysed with lysis buffer and lysate (cell lysate) was used for the estimation of antioxidant enzymes: catalase, superoxide dismutase, glutathione-S-transferase, glutathione peroxidase as described previously [2], [48]–[55]. Lipid peroxides {(as malondialdehyde (MDA) formed on reaction with thiobarbituric acid (TBA)} and nitric oxide {(NO), measured as nitrite formed using Griess reagent)} were estimated both in the supernatant and cell lysate as described previously [2], [50]–[52].

### Statistical Analysis

Data obtained were analysed by paired t-test using MS-Excel statistical analysis tool. Each treatment was repeated twice on different occasions in triplicate. The values are presented as mean ± SEM.

## Results

### Effect of bleomycin on the proliferation of IMR-32 cells *in vitro*

**Figure 1:**
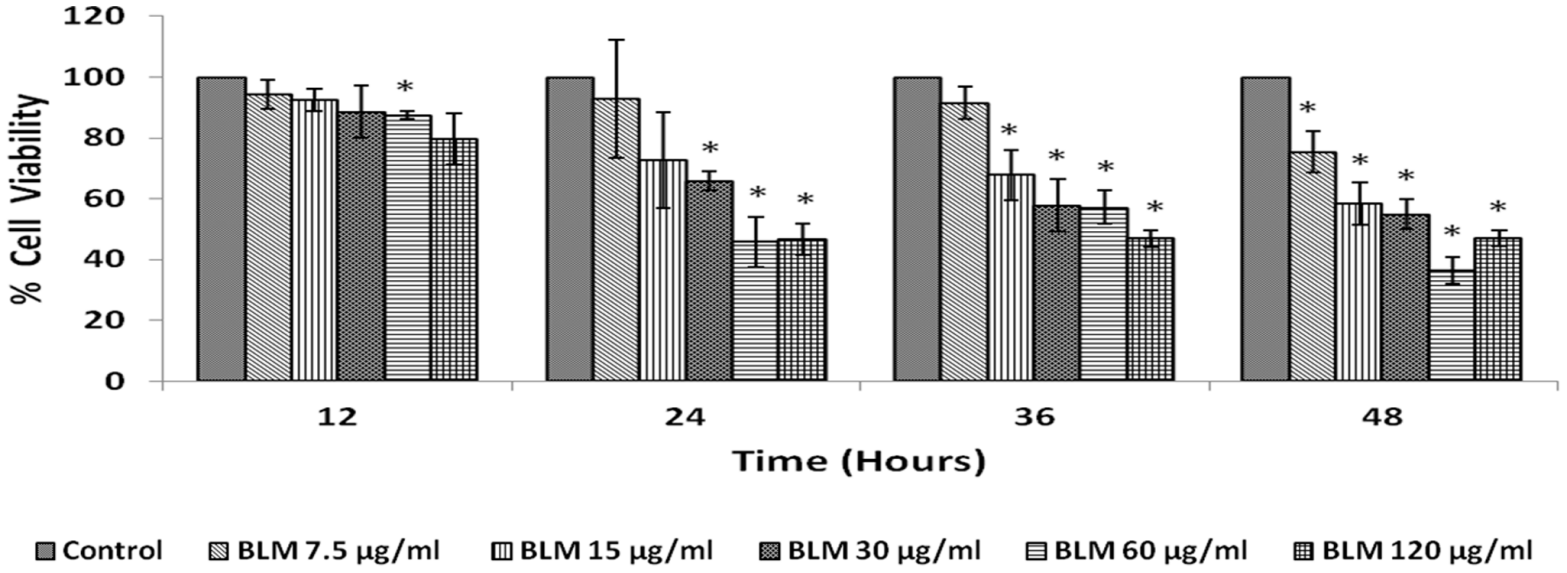
Dose and time optimization of bleomycin. IMR-32 cells were exposed to different doses of bleomycin (7.5–120 µg/ml) and incubated for 12–48 hrs. At the end of treatment period, cell viability was measured by MTT assay. All values are expressed as mean ± standard error (n = 6). *P<0.05 compared to control. BLM – Bleomycin.

The results of this study given in Fig. 1 showed that IMR-32 cells when exposed to different concentration of bleomycin (7.5−120 µg/ml) for different periods of time (12−48 hrs), 60 µg/ml decreased their proliferation by ∼50% at the end of 24 hrs of incubation (Fig. 1). Hence, all further studies were done using 60 µg/ml.

### Effect of various PUFAs and its metabolites on the proliferation of IMR-32 cells *in vitro*

#### Effect of PUFAs

**Figure 2:**
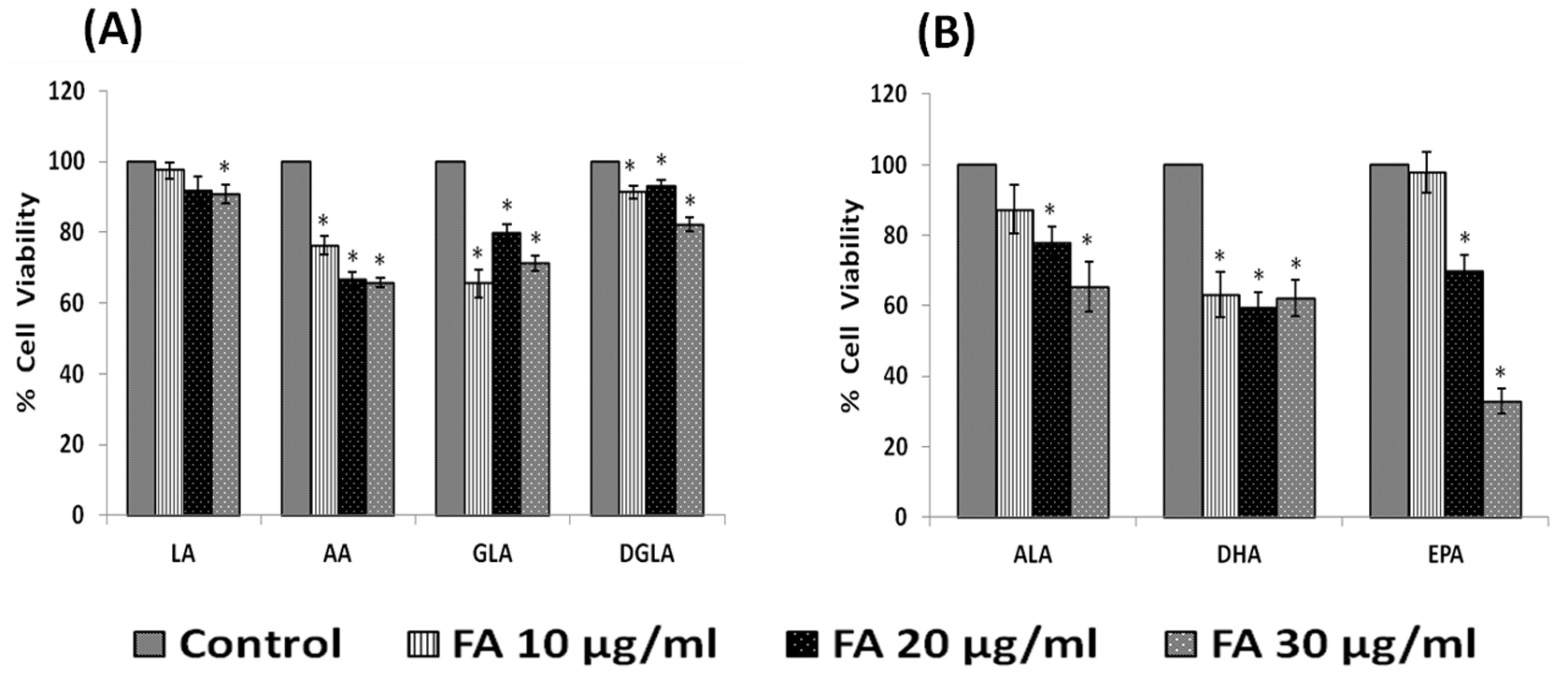
Effect of PUFAs on IMR-32 cells. IMR-32 cells were exposed to different doses (10, 20, 30 µg/ml) of n-6 (Fig. 2A) and n-3 (Fig. 2B) fatty acids and incubated for 24 hrs. At the end of treatment period, cell viability was measured by MTT assay. All values are expressed as mean ± standard error (n = 6). *P<0.05 when compared to control. LA – Linoleic Acid, AA – Arachidonic acid, GLA – Gamma Linoleic Acid, DGLA – Dihomo Gamma Linoleic Acid, ALA – Alpha Linoleic Acid, DHA – Docosahexaenoic Acid, EPA – Eicosapentaenoic Acid.

The effect of various PUFAs: LA, AA, GLA, DGLA, ALA, EPA and DHA on the proliferation of IMR-32 cells when exposed to 10, 20, 30 µg/ml for 24 hours revealed that all the PUFAs tested are able to decrease their proliferation to a significant degree (p<0.001) compared to control (Figs. 2A and 2B).

#### Effect of lipoxin A_4_

**Figure 3:**
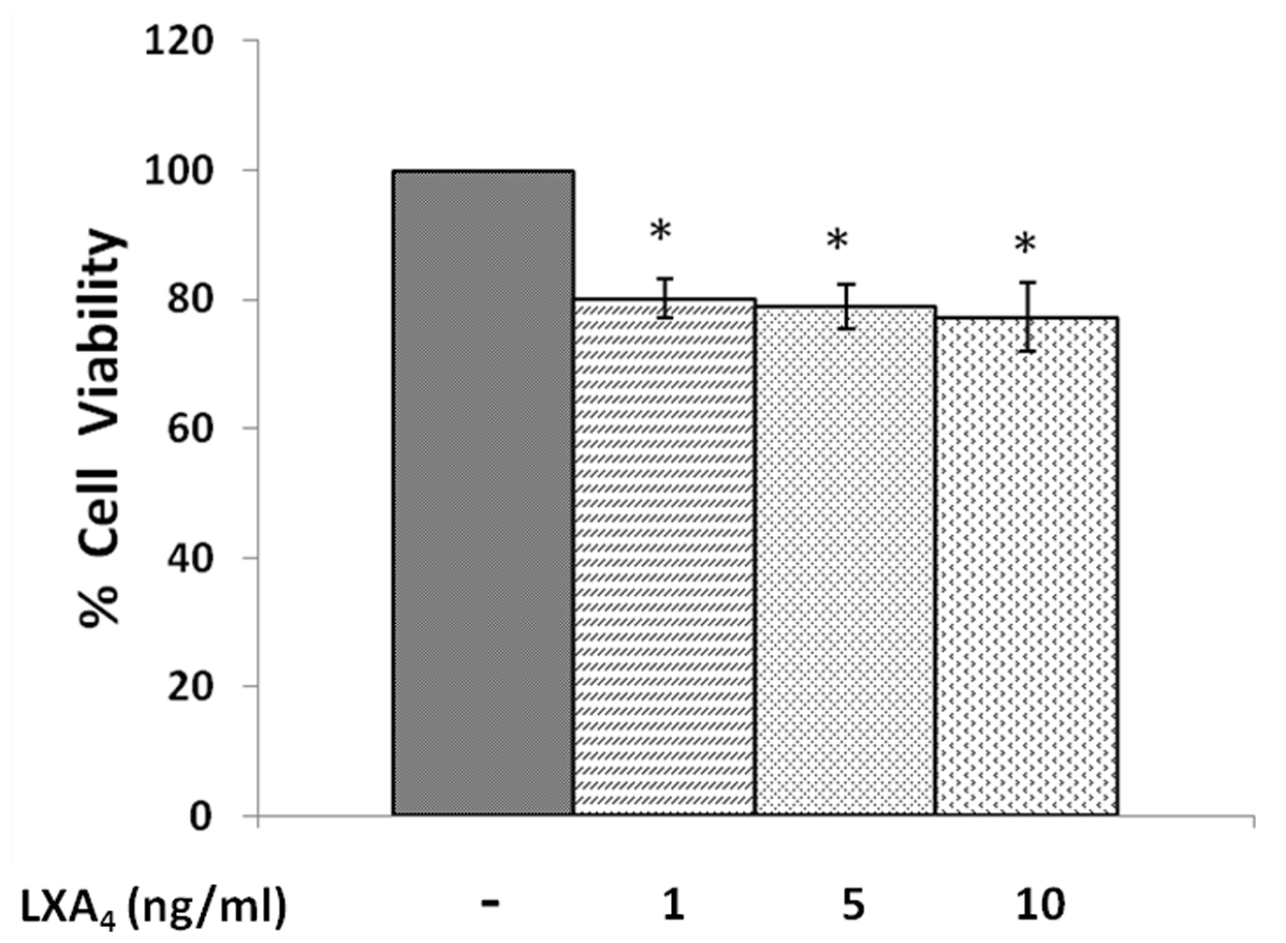
Effect of lipoxin A4 on IMR-32 cells. IMR-32 cells were exposed to different doses (1, 5, 10 ng/ml) of lipoxin A4 and incubated for 24 hrs. At the end of treatment period, cell viability was measured by MTT assay. All values are expressed as mean ± standard error (n = 6). *P<0.05 when compared to control. LXA_4_– Lipoxin A_4_.

When IMR-32 cells were exposed to different doses of lipoxin A4 (1, 5, 10 ng/ml) and incubated for 24 hrs, a significant reduction in the proliferation of the cells was noted (p<0.001) in a dose dependent manner compared to control (Fig. 3).

#### Effect of resolvins

**Figure 4:**
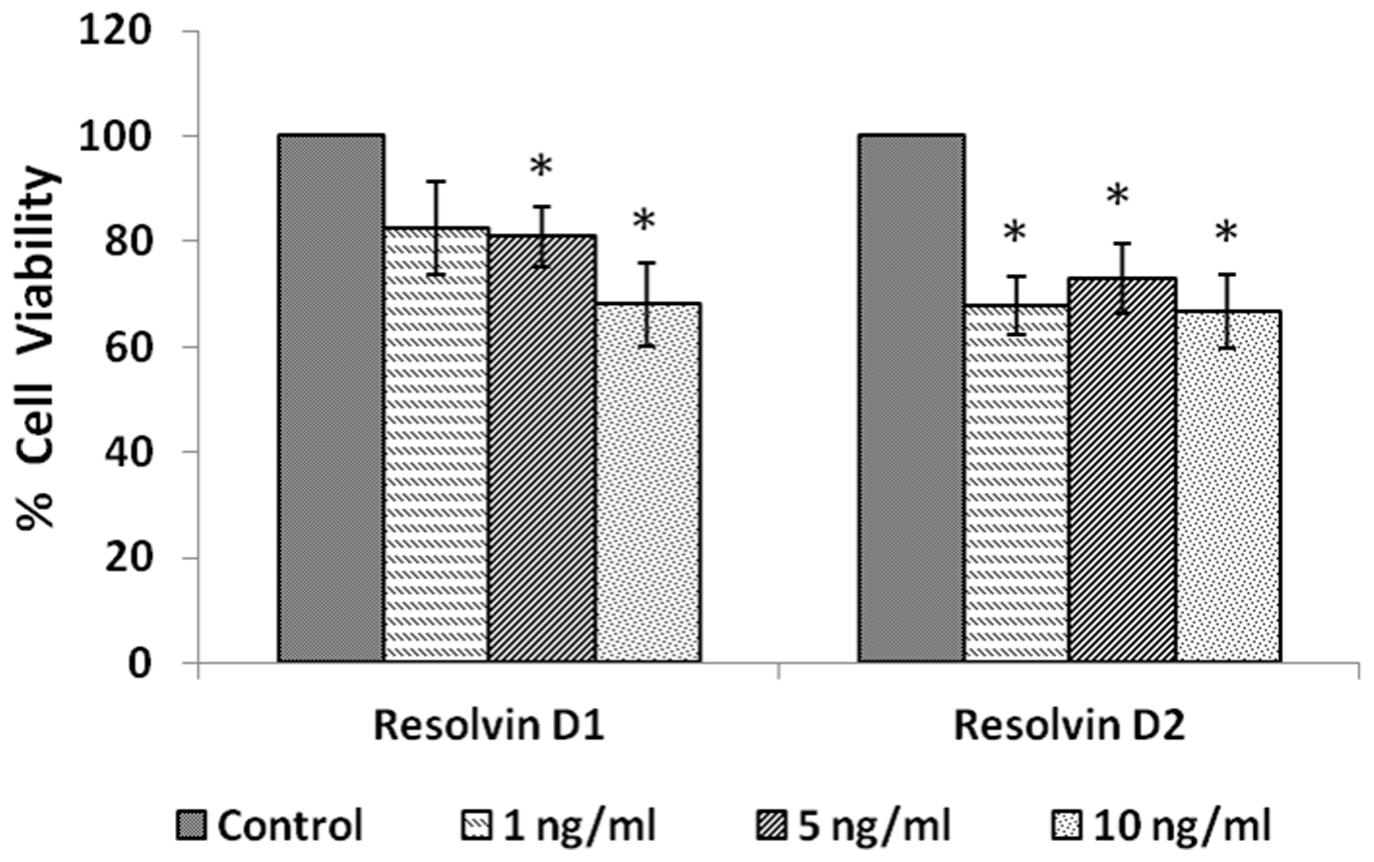
Effect of resolvin on IMR-32 cells. IMR-32 cells were exposed to different doses (1, 5, 10 ng/ml) of resolvin (D1, D2) and incubated for 24 hrs. At the end of treatment period, cell viability was measured by MTT assay. All values are expressed as mean ± standard error (n = 6). *P<0.05 when compared to control.

IMR-32 cells, when were exposed to different concentrations of resolvins (1, 5, 10 ng/ml), both resolvin D1 and D2 significantly (p<0.01) decreased the proliferation of the cells (Fig. 4) compared to control.

#### Effect of protectins

**Figure 5:**
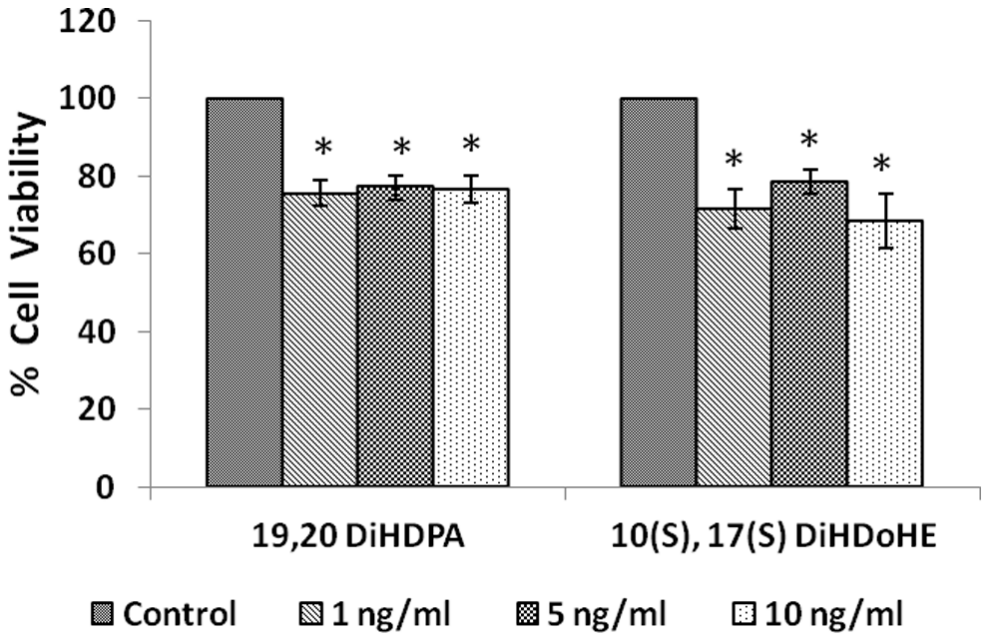
Effect of protectin on IMR-32 cells. IMR-32 cells were exposed to different doses (1, 5, 10 ng/ml) of protectin (19,20-DiHDPA, 10(S),17(S)-DiHDoHE) and incubated for 24 hrs. At the end of treatment period, cell viability was measured by MTT assay. All values are expressed as mean ± standard error (n = 6). *P<0.05 when compared to control.

IMR-32 cells, when exposed to different concentrations (1, 5, 10 ng/ml) of protectins (19,20-DiHDPA and 10(S),17(S) DiHDoHE), a significant reduction in the proliferation of cells was noted compared to control (p<0.01) (Fig. 5).

#### Effect of prostaglandins

**Figure 6:**
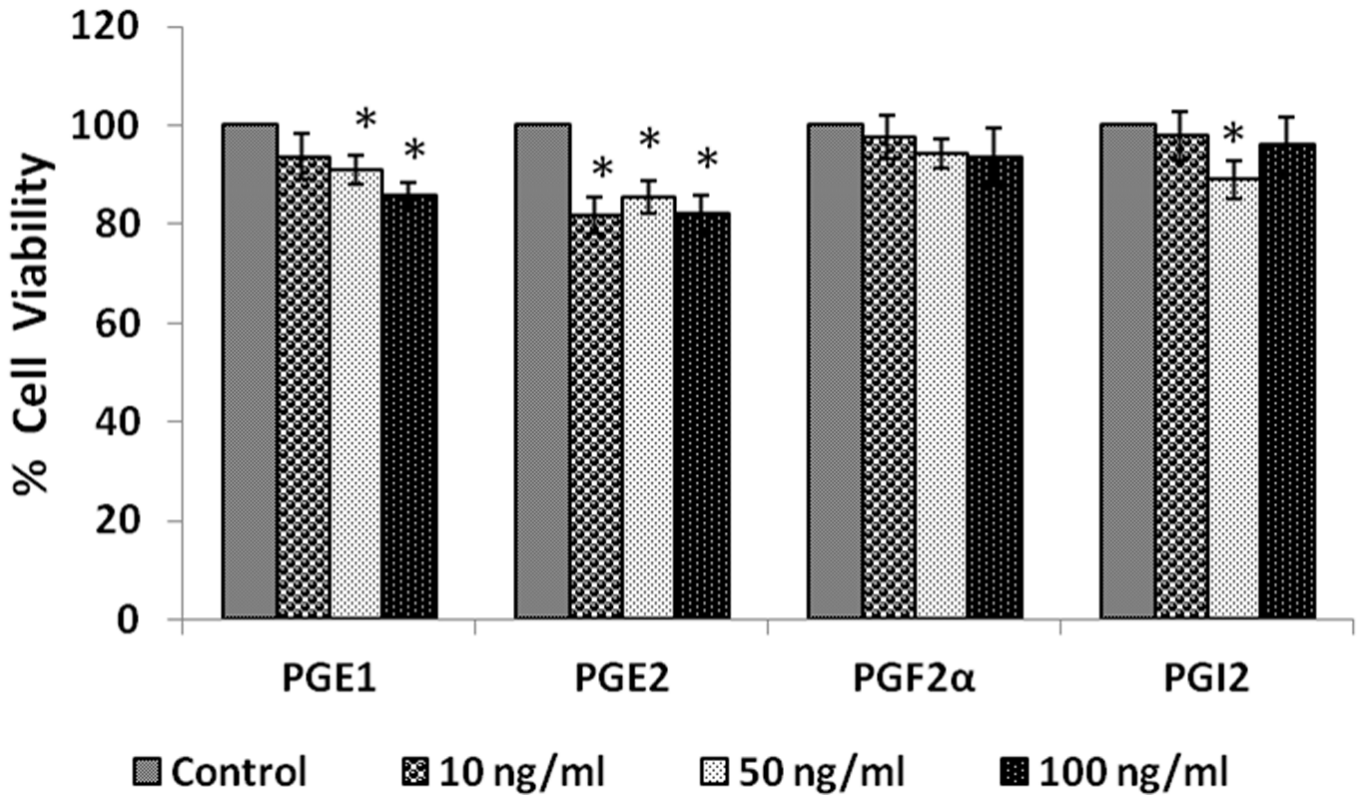
Effect of prostaglandin on IMR-32 cells. IMR-32 cells were exposed to different doses (10, 50, 100 ng/ml) of prostaglandins (PGE1, PGE2, PGF2α, PGI2) and incubated for 24 hrs. At the end of treatment period, cell viability was measured by MTT assay. All values are expressed as mean ± standard error (n = 6). *P<0.05 when compared to control. PG = Prostaglandin.

When IMR-32 cells were exposed to different doses (10, 50, 100 ng/ml) of various prostaglandins: PGE1, PGE2, PGF2α, PGI2 for 24 hours; only E_1_ and E_2_ induced a significant reduction in the proliferation (p<0.05) of the cells (Fig. 6).

#### Effect of leukotrienes

**Figure 7:**
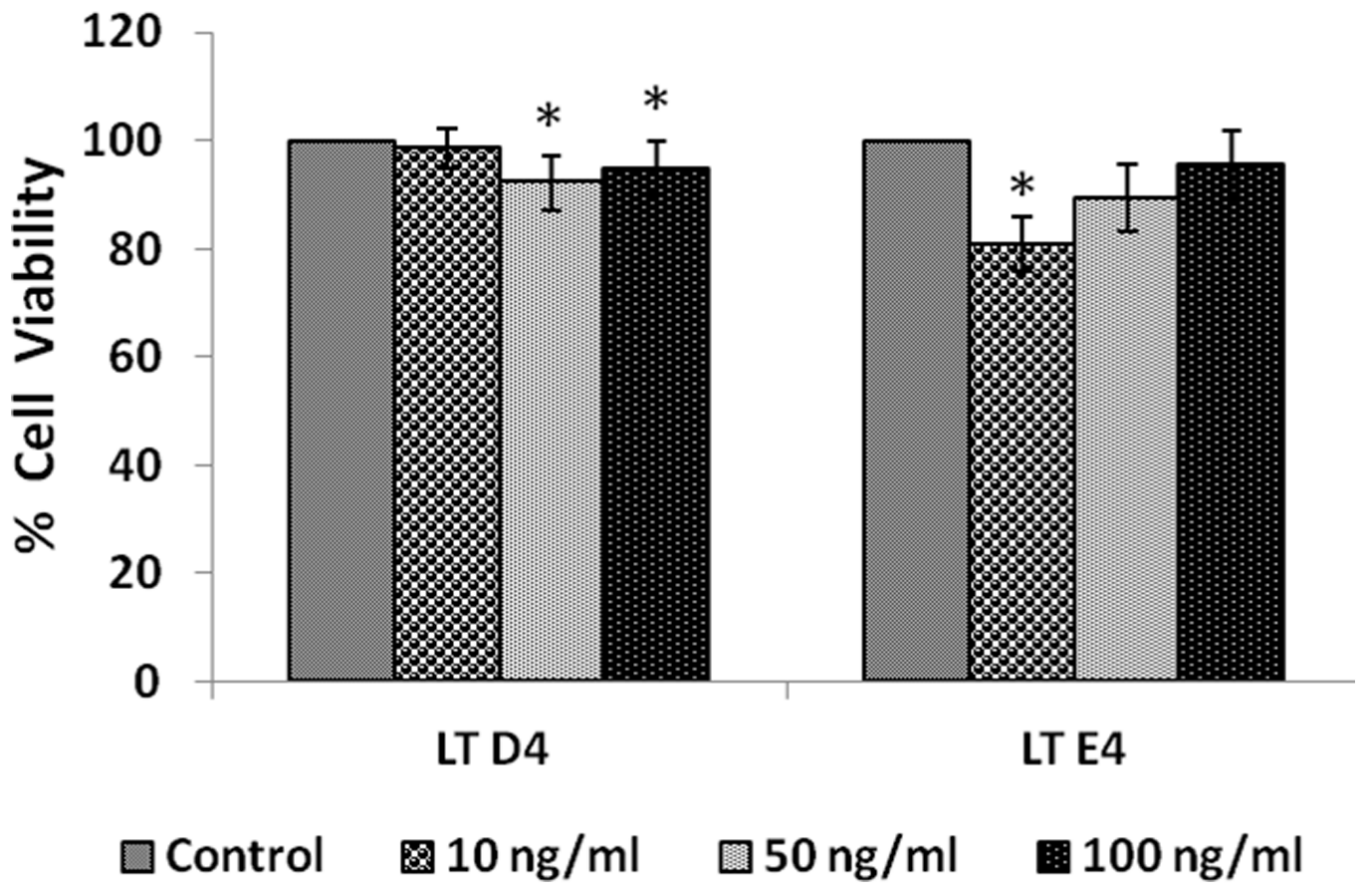
Effect of leukotriene on IMR-32 cells. IMR-32 cells were exposed to different doses (10, 50, 100 ng/ml) of leukotrienes (D4, E4) and incubated for 24 hrs. At the end of treatment period, cell viability was measured by MTT assay. All values are expressed as mean ± standard error (n = 6). *P<0.05 when compared to control. LT = Leukotriene.

IMR-32 cells when treated with different doses (10, 50, 100 ng/ml) of leukotrienes LTD4 and LTE4 for 24 hours, LTD4 was more effective than LTE4 in inducing significant inhibition in the proliferation of the cells (Fig. 7, P<0.01) when compared to control.

#### Effect of various PUFAs and their metabolites on bleomycin induced suppression of proliferation of IMR-32 cells *in vitro*

In order to know whether PUFAs and their various metabolites alter the growth inhibitory action of bleomycin on IMR-32 cells *in vitro*, we studied the effect pre- and simultaneous exposure of these cells to PUFAs, prostaglandins, leukotrienes, lipoxin A4, resolvins and protectins and bleomycin.

#### Effect of PUFAs

**Figure 8:**
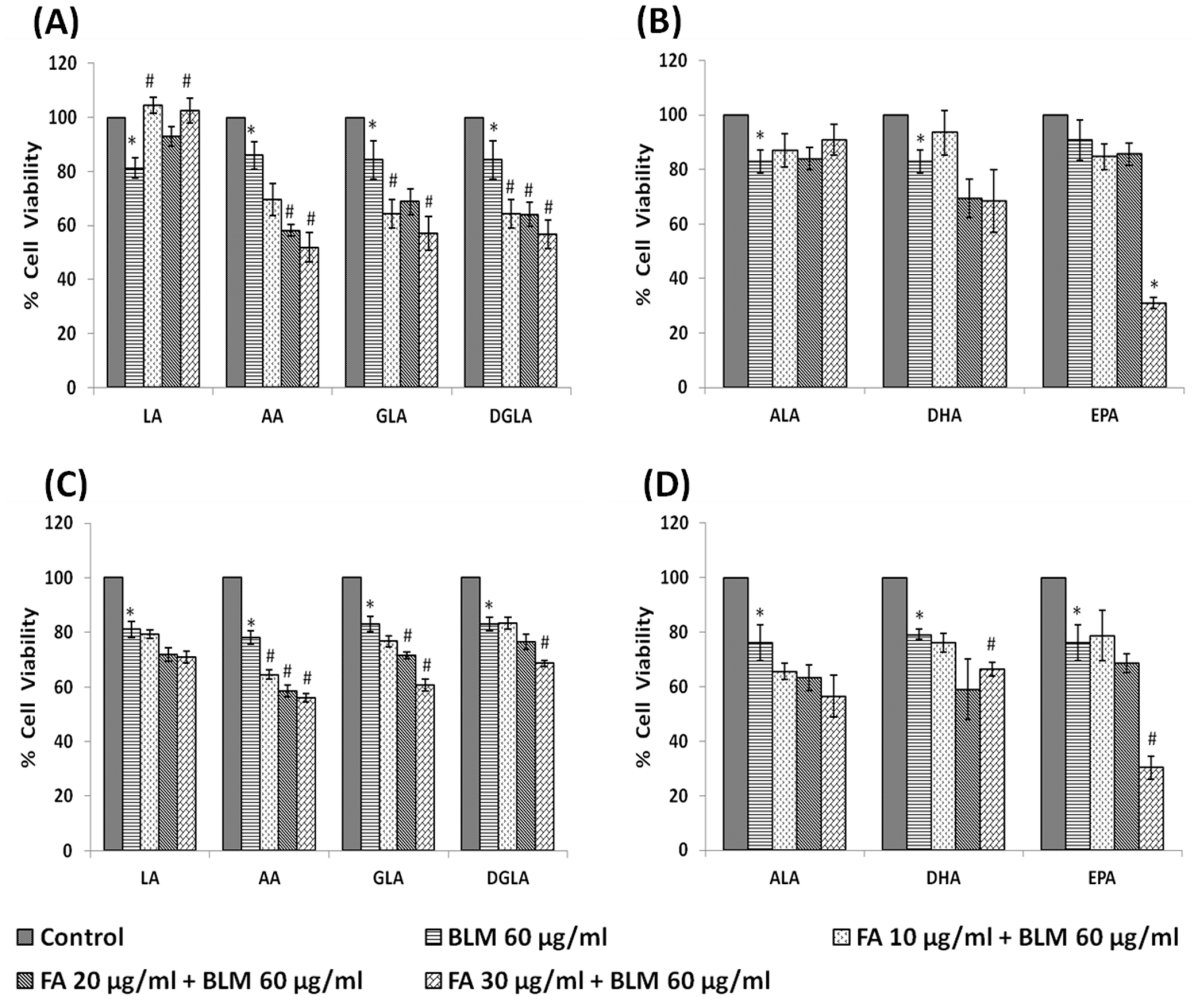
Effect of PUFAs on bleomycin induced cytotoxicity in IMR-32 cells. Effect of pre-treatment with different doses (10, 20, 30 µg/ml) of n-6 (Fig. 8A) and n-3 (Fig. 8B) PUFAs on bleomycin (60 µg/ml) induced cytotoxicity to IMR-32 cells. Cells were pre incubated with PUFAs for 5 hrs and then bleomycin for 24 hrs. MTT assay was performed. Effect of simultaneous treatment with different doses (10, 20, 30 µg/ml) of n-6 (Fig. 8C) and n-3 (Fig. 8D) PUFAs on bleomycin (60 µg/ml) induced cytotoxicity to IMR-32 cells. Cells were pre incubated with plain media for 5 hrs and then PUFAs and bleomycin were added and incubated for 24 hrs. MTT assay was performed. All values are expressed as mean ± standard error (n = 6). *P<0.05 when compared to control; #P<0.05 when compared to bleomycin. LA = Linoleic Acid, AA = Arachidonic acid, GLA = Gamma Linoleic Acid, DGLA = Dihomo Gamma Linoleic Acid, ALA = Alpha Linoleic Acid, DHA = Docosahexaenoic Acid, EPA = Eicosapentaenoic Acid.

In the pre-treatment studies, IMR-32 cells were first exposed to PUFAs for 5 hrs following which the medium was replaced with fresh media containing bleomycin (60 µg/ml) and incubated for an additional 24 hrs. At the end of the treatment period, viable cell numbers were measured by MTT assay. The results of this study given in Fig. 8, revealed that GLA, DGLA, AA and EPA significantly (p<0.05) enhanced bleomycin-induced growth inhibitory action on IMR-32 cells in both pre- and simultaneous treatment schedules. Of all the PUFAs tested, AA was the most effective in enhancing the growth inhibitory action of bleomycin on IMR-32 cells. Hence, all further experiments in the present study focused on the effects of arachidonic and bleomycin on IMR-32 cells.

#### Effect of lipoxin A_4_, resolvins, protectins and prostaglandins

**Figure 9:**
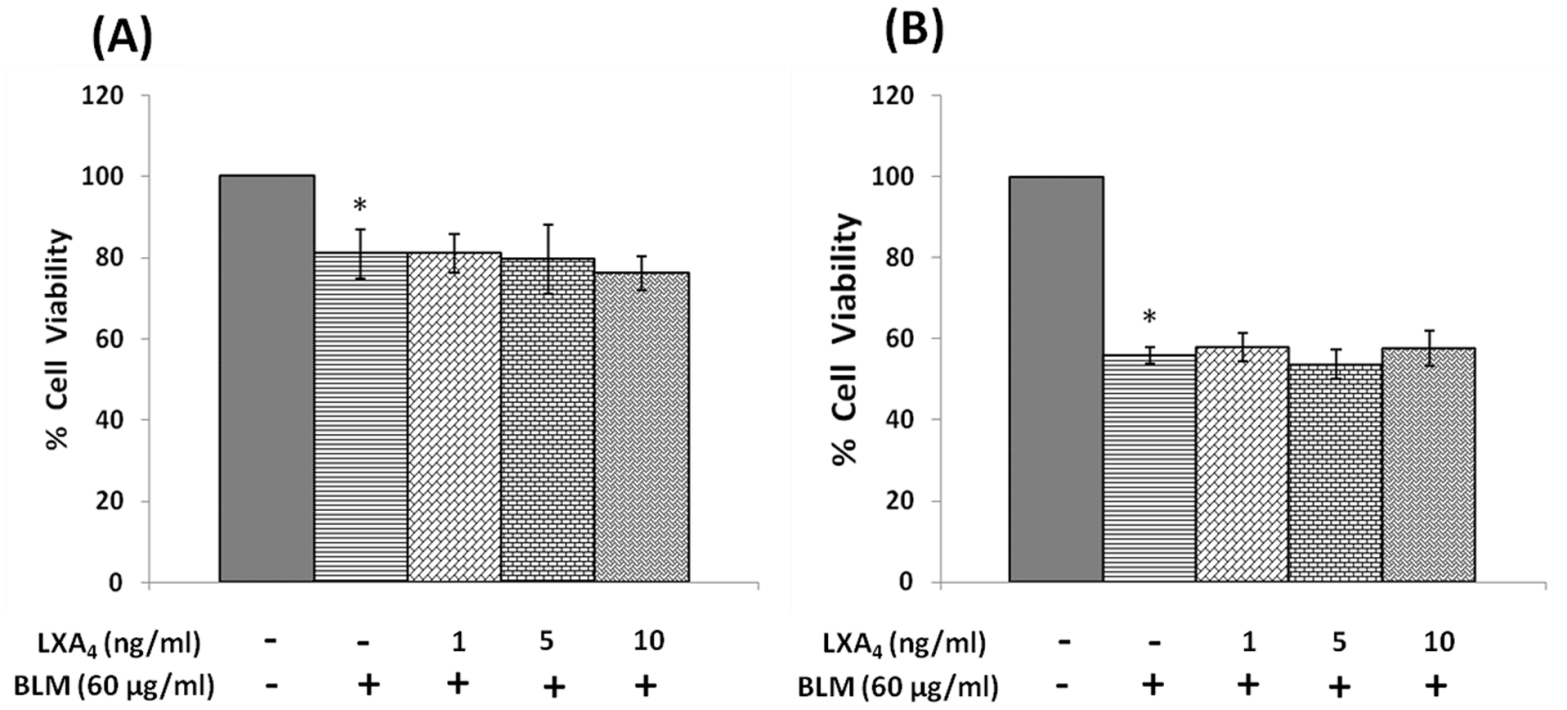
Effect of Lipoxin A4 on bleomycin induced cytotoxicity in IMR-32 cells. (A) Effect of pre-treatment with different doses (1, 5, 10 ng/ml) of lipoxin A4 on bleomycin (60 µg/ml) induced cytotoxicity to IMR-32 cells. Cells were pre incubated with lipoxin A4 for 5 hrs and then bleomycin for 24 hrs. MTT assay was performed. (B) Effect of simultaneous treatment with different doses (1, 5, 10 ng/ml) of lipoxin A4 on bleomycin (60 µg/ml) induced cytotoxicity to IMR-32 cells. Cells were pre incubated with plain media for 5 hrs and then lipoxin A4 and bleomycin were added and incubated for 24 hrs. MTT assay was performed. All values are expressed as mean ± standard error (n = 6). *P<0.05 when compared to control; #P<0.05 when compared to bleomycin. BLM = Bleomycin, LXA_4_ = Lipoxin A_4_.

**Figure 10:**
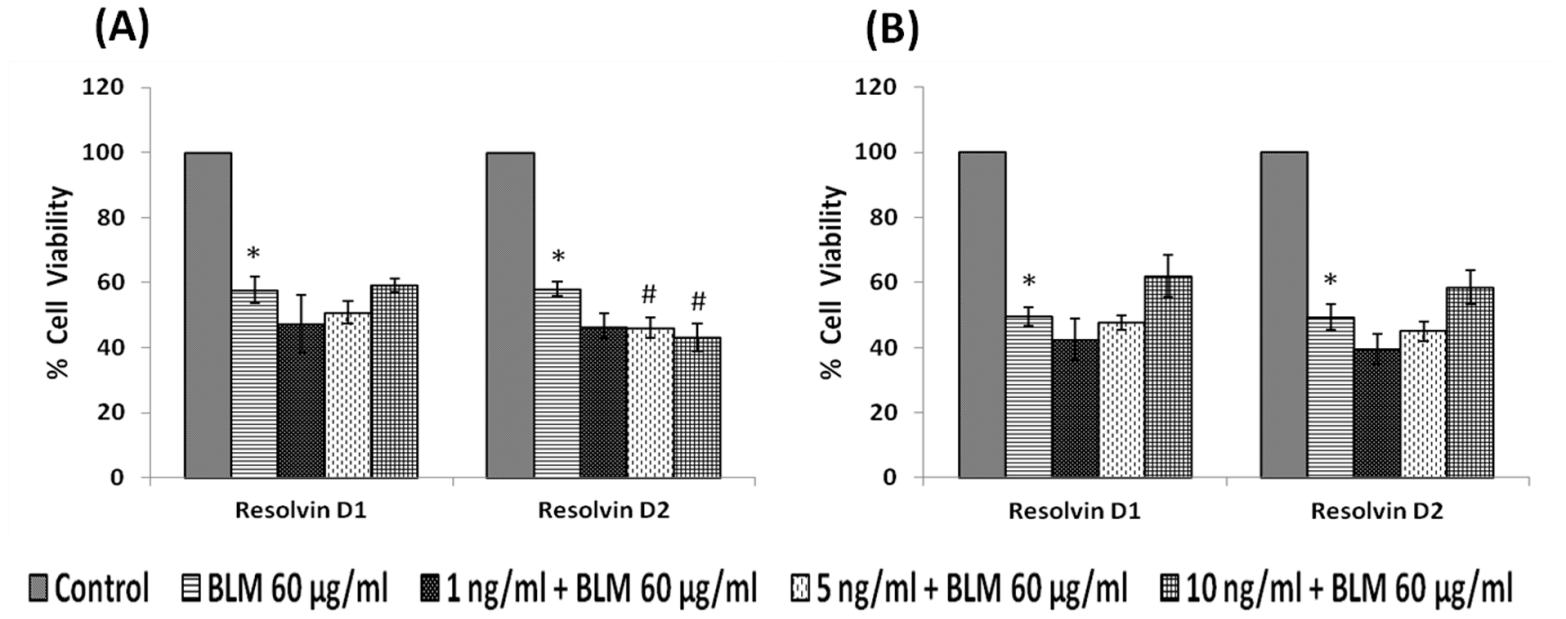
Effect of resolvin on bleomycin induced cytotoxicity in IMR-32 cells. (A) Effect of pre-treatment with different doses (1, 5, 10 ng/ml) of resolvin (D1, D2) on bleomycin (60 µg/ml) induced cytotoxicity to IMR-32 cells. Cells were pre incubated with resolvin for 5 hrs and then bleomycin for 24 hrs. MTT assay was performed. (B) Effect of simultaneous treatment with different doses (1, 5, 10 ng/ml) of resolvin (D1, D2) on bleomycin (60 µg/ml)-induced cytotoxicity to IMR-32 cells. Cells were pre incubated with plain media for 5 hrs and then resolvin and bleomycin were added and incubated for 24 hrs. MTT assay was performed. All values are expressed as mean ± standard error (n = 6). *P<0.05 when compared to control; #P<0.05 when compared to bleomycin. BLM = Bleomycin.

**Figure 11:**
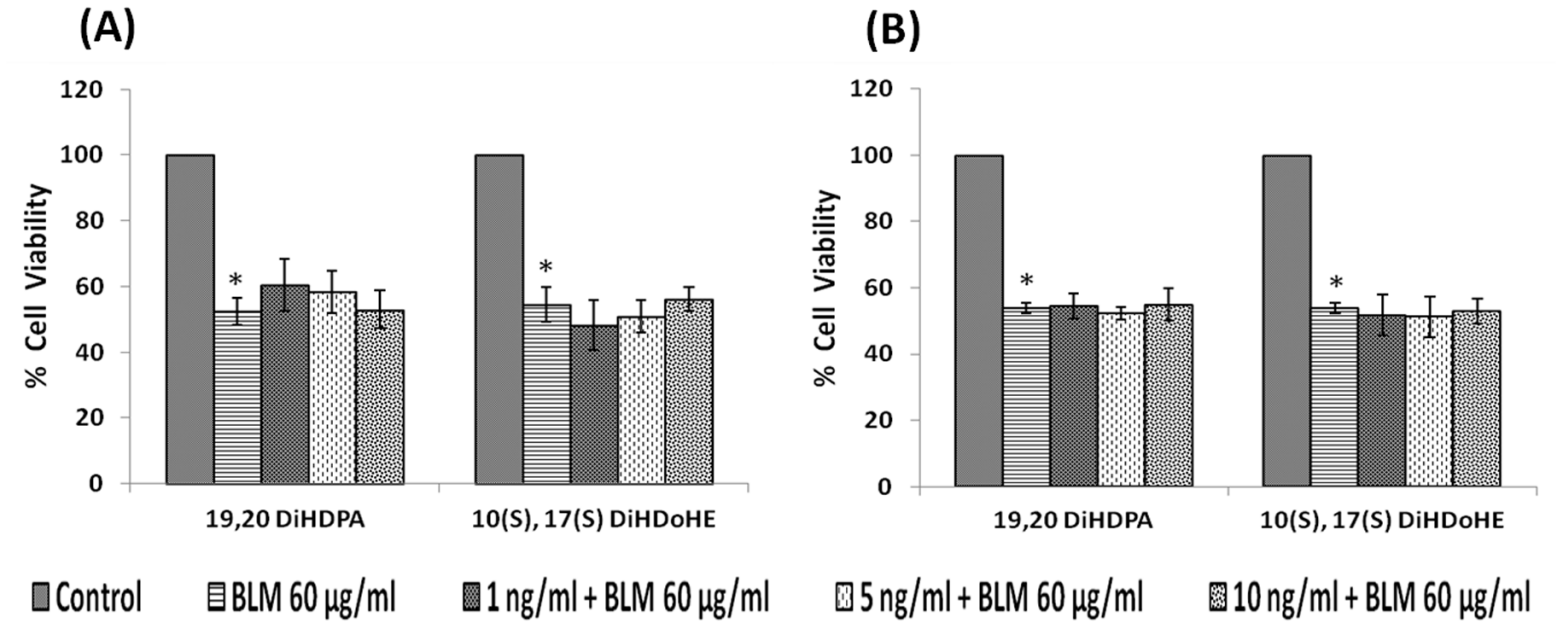
Effect of protectin on bleomycin induced cytotoxicity in IMR-32 cells. (A) Effect of pre-treatment with different doses (1, 5, 10 ng/ml) of protectin (19,20-DiHDPA, 10(S),17(S)-DiHDoHE) on bleomycin (60 µg/ml) induced cytotoxicity to IMR-32 cells. Cells were pre incubated with protectin for 5 hrs and then bleomycin for 24 hrs. MTT assay was performed. (B) Effect of simultaneous treatment with different doses (1, 5, 10 ng/ml) of protectin (19,20-DiHDPA, 10(S),17(S)-DiHDoHE) on bleomycin (60 µg/ml) induced cytotoxicity to IMR-32 cells. Cells were pre incubated with plain media for 5 hrs and then protectin and bleomycin were added and incubated for 24 hrs. MTT assay was performed. All values are expressed as mean ± standard error (n = 6). *P<0.05 when compared to control. BLM – Bleomycin.

Both pre- and simultaneous treatment with lipoxin A4 did not produce any significant change in the growth inhibitory action of bleomycin on IMR-32 cells *in vitro* (Fig. 9). Similarly, both resolvins and protectins also did not produce any significant change in the growth inhibitory action of bleomycin on IMR-32 cells *in vitro* (Figs. 10 and 11).

**Figure 12:**
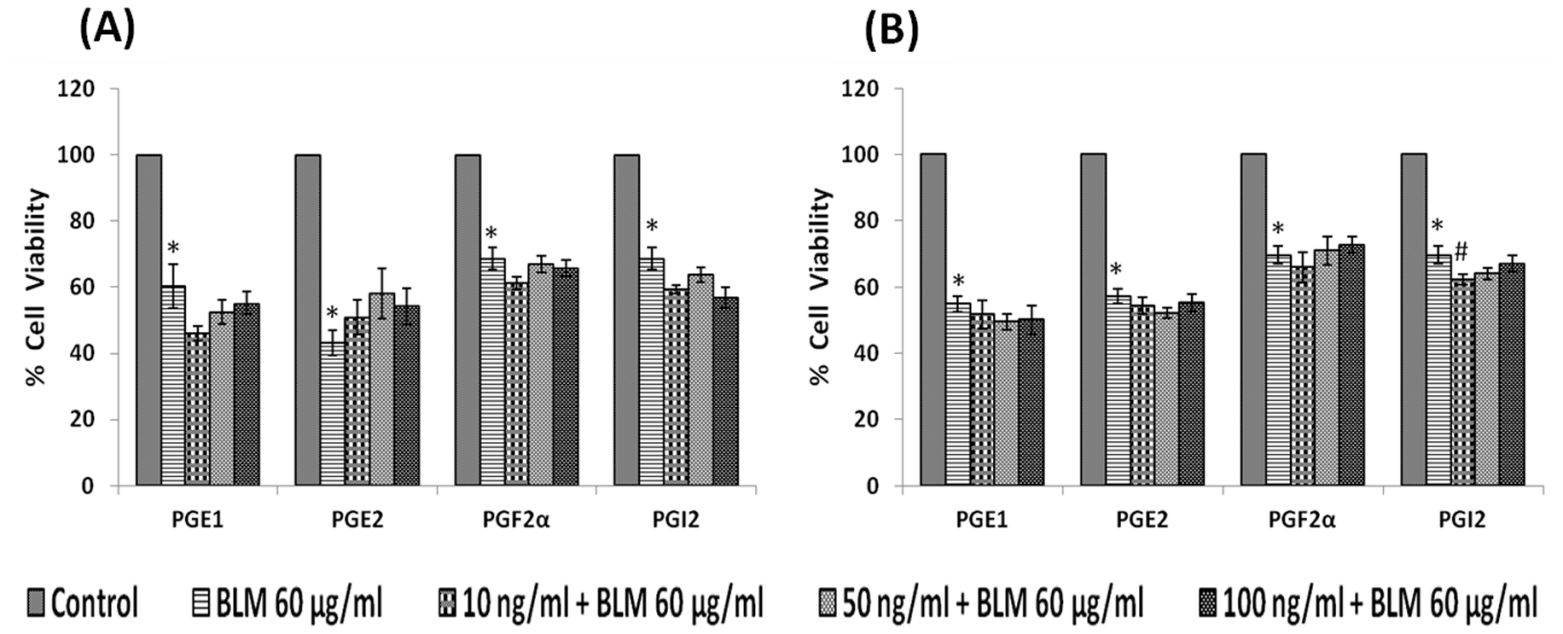
Effect of prostaglandins on bleomycin induced cytotoxicity in IMR-32 cells. (A) Effect of pre-treatment with different doses (10, 50, 100 ng/ml) of prostaglandins (PGE1, PGE2, PGF2α, PGI2) on bleomycin (60 µg/ml)- induced cytotoxicity to IMR-32 cells. Cells were pre incubated with prostaglandin for 5 hrs and then bleomycin for 24 hrs. MTT assay was performed. (B) Effect of simultaneous treatment with different doses (10, 50, 100 ng/ml) of prostaglandin (PGE1, PGE2, PGF2α, PGI2) on bleomycin (60 µg/ml) induced cytotoxicity to IMR-32 cells. Cells were pre incubated with plain media for 5 hrs and then prostaglandin and bleomycin were added and incubated for 24 hrs. MTT assay was performed. All values are expressed as mean ± standard error (n = 6). *P<0.05 when compared to control; #P<0.05 when compared to bleomycin. BLM = Bleomycin, PG = Prostaglandin.

**Figure 13:**
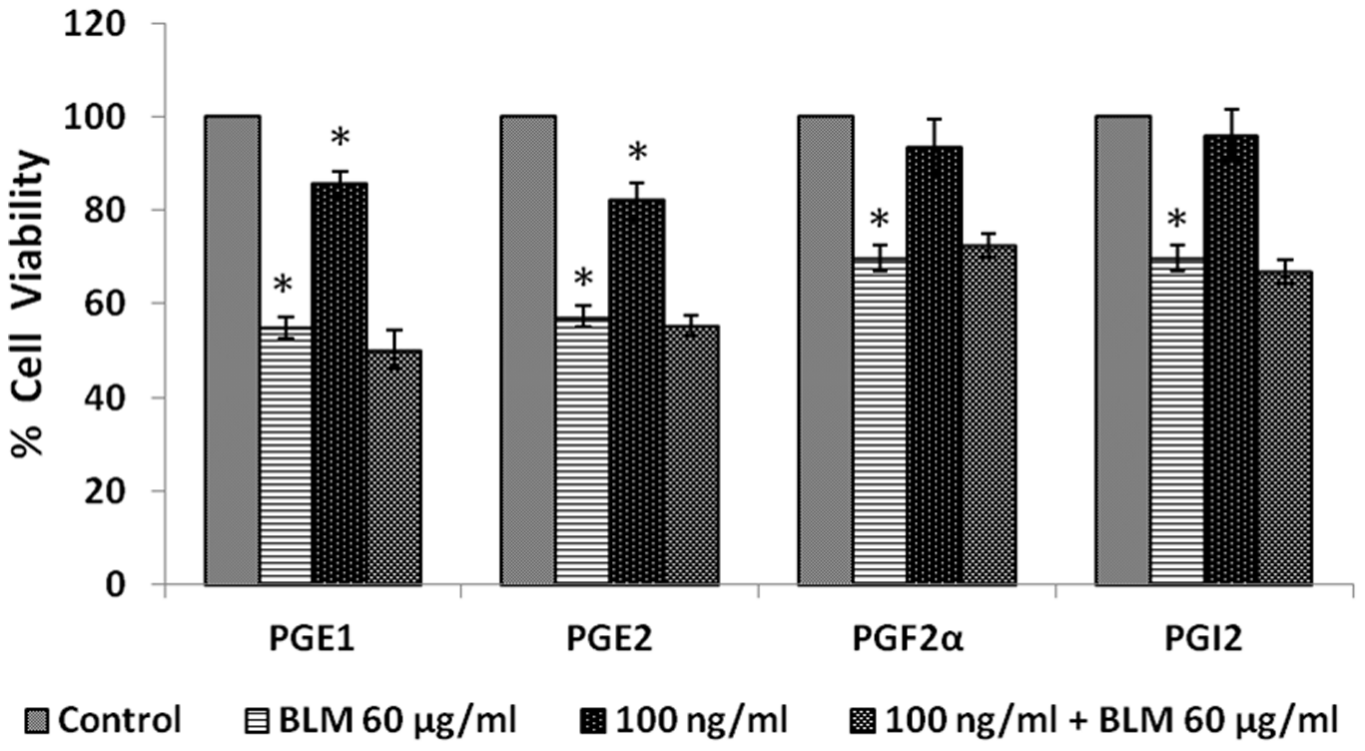
Effect of prostaglandin on bleomycin induced cytotoxicity in IMR-32 cells. Effect of simultaneous treatment with 100 ng/ml dose of prostaglandins (PGE1, PGE2, PGF2α, PGI2) on bleomycin (60 µg/ml)-induced cytotoxicity to IMR-32 cells. Cells were pre incubated with plain media for 5 hrs and then prostaglandin and bleomycin were added together and incubated for 24 hrs. MTT assay was performed. All values are expressed as mean ± standard error (n = 6). *P<0.05 when compared to control. BLM = Bleomycin, PG = Prostaglandin. It can be seen that PGs themselves did not have significant inhibitory action on the growth of IMR-32 cells except for PGE1 and PGE2 that showed inhibitory action on the growth of IMR-32 cells, while PGF2α and PGI2 were without any inhibitory action.

Similar to lipoxin A4, resolvins and protectins, even prostaglandins (PGE1, PGE2, PGF2α and PGI2) tested both in the pre- and simultaneous treatment schedules did not influence the growth inhibitory action of bleomycin on IMR-32 cells *in vitro*, though in the simultaneous treatment schedule PGI2 enhanced the growth inhibitory action of bleomycin (Fig. 12). It is evident that PGs themselves did not have significant inhibitory action on the growth of IMR-32 cells except for PGE1 and PGE2 that showed inhibitory action on the growth of IMR-32 cells in the simultaneous treatment group (Fig. 13), while PGF2α and PGI2 were without any inhibitory action.

#### Effect of leukotrienes

**Figure 14:**
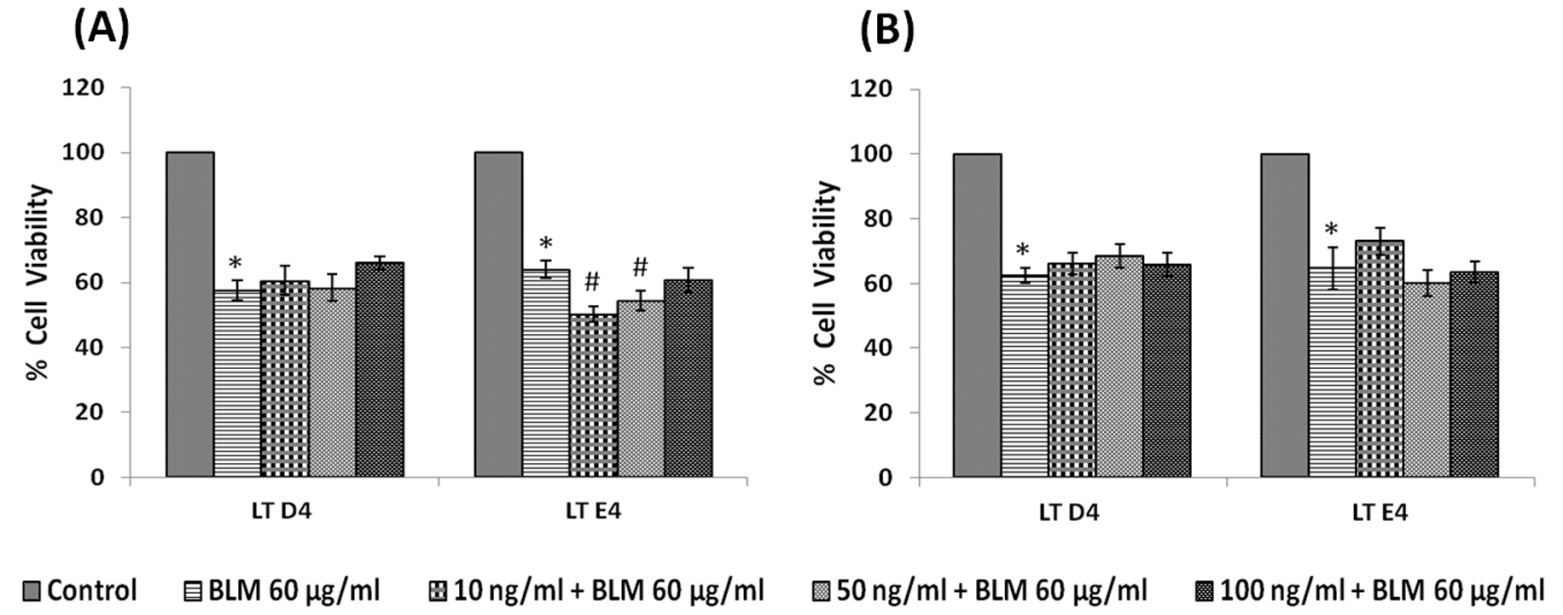
Effect of Leukotriene on bleomycin induced cytotoxicity in IMR-32 cells. (A) Effect of pre-treatment with different doses (10, 50, 100 ng/ml) of leukotriene (LTD4, LTE4) on bleomycin (60 µg/ml) induced cytotoxicity to IMR-32 cells. Cells were pre incubated with leukotriene for 5 hrs and then bleomycin for 24 hrs. MTT assay was performed. (B) Effect of simultaneous treatment with different doses (10, 50, 100 ng/ml) of leukotriene (LTD4, LTE4) on bleomycin (60 µg/ml) induced cytotoxicity to IMR-32 cells. Cells were pre incubated with plain media for 5 hrs and then leukotriene and bleomycin were added and incubated for 24 hrs. MTT assay was performed. All values are expressed as mean ± standard error (n = 6). *P<0.05 when compared to control; #P<0.05 when compared to bleomycin. BLM – Bleomycin, LT - Leukotriene.

Both pre- and simultaneous treatment schedules with LTD4 and LTE4 did not show any significant effect on the growth inhibitory action of bleomycin on IMR-32 cells *in vitro*, though pre-treatment with 10 and 50 ng of LTE4 potentiated the growth inhibitory action of bleomycin (Fig. 14).

#### Effect of COX and LOX inhibitors

**Figure 15:**
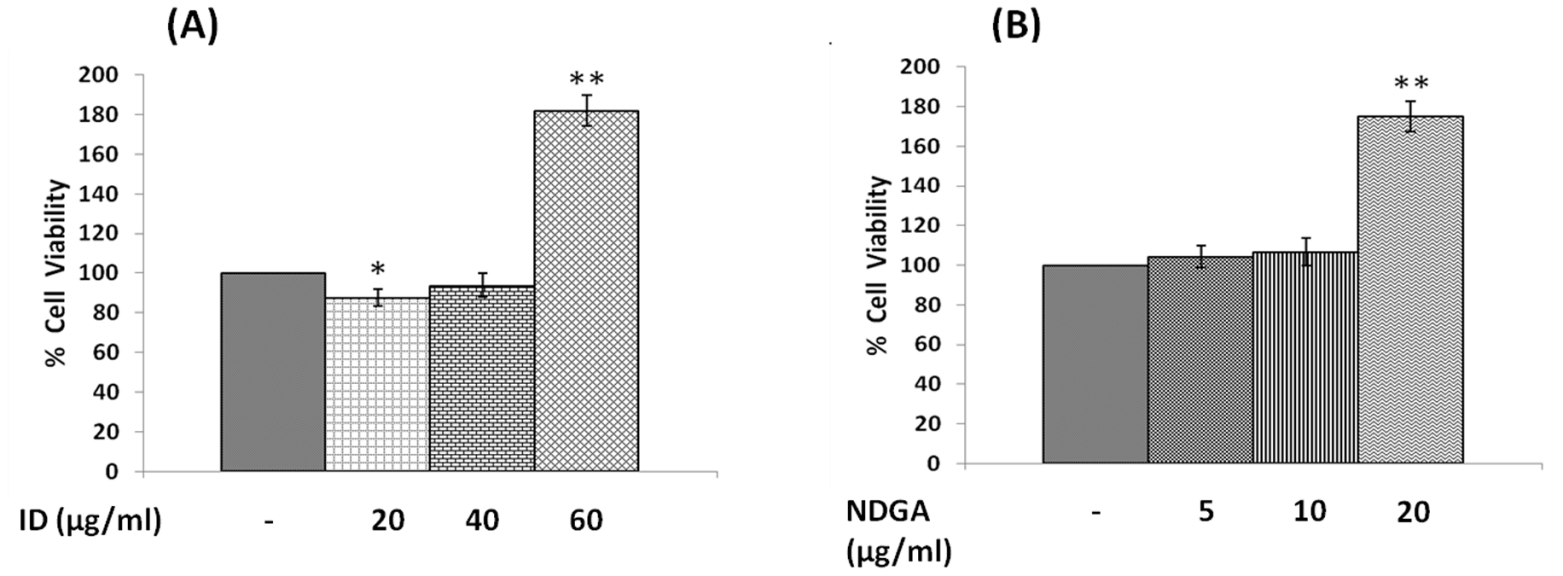
Effect of COX and LOX inhibitor on IMR-32 cells. (A) Effect of indomethacin (20, 40, 60 µg/ml) on viability of IMR-32 cells during 24 hrs incubation. (B) Effect of NDGA (5, 10, 20 µg/ml) on viability of IMR-32 cells during 24 hrs incubation. All values are expressed as mean ± standard error (n = 6). *P<0.05 when compared to control. ID = Indomethacin, NDGA = Nordihydroguariaretic acid.

Since prostaglandins, lipoxin A4, resolvins and protectins did not enhance, whereas PUFAs enhanced the growth inhibitory action of bleomycin, we next studied the effect of COX and LOX inhibitors indomethacin and NDGA respectively on the growth of IMR-32 cells. IMR-32 cells were treated with indomethacin, a COX inhibitor (20, 40, 60 µg/ml) and NDGA, a LOX inhibitor (5, 10, 20 µg/ml). To our surprise, it was found that both indomethacin (60 µg/ml) and NDGA (20 µg/ml) significantly enhanced (p<0.001) IMR-32 cell proliferation compared to control (Fig. 15).

#### Effect of cyclo-oxygenase (COX) and lipoxygenase (LOX) inhibitors on AA and bleomycin induced growth inhibitory action on IMR-32 cells

**Figure 16:**
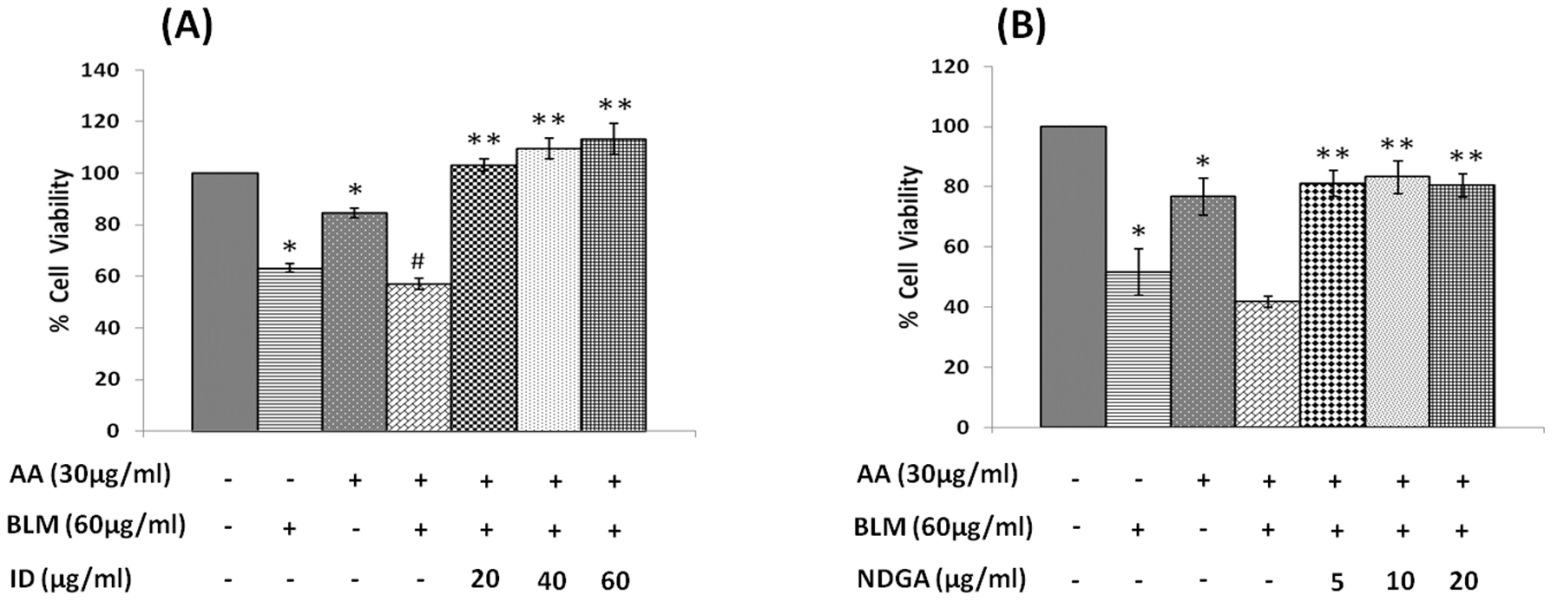
Effect of COX and LOX inhibitors on arachidonic acid mediated action on bleomycin induced cytotoxicity on IMR-32 cells. (A) Effect of Indomethacin (20, 40, 60 µg/ml) on arachidonic acid (30 µg/ml)+bleomycin action (60 µg/ml) on IMR-32 cells during 24 hrs incubation. (B) Effect of NDGA (5, 10, 20 µg/ml) on arachidonic acid (30 µg/ml)+bleomycin action (60 µg/ml) on IMR-32 cells during 24 hrs incubation. All values are expressed as mean ± standard error (n = 6). *P<0.05 when compared to control. **P<0.001 when compared to AA+BLM. #P<0.05 when compared with bleomycin. AA = Arachidonic Acid, BLM = Bleomycin, ID = Indomethacin, NDGA = Nordihydroguariaretic acid.

To explore further the role of indomethacin and NDGA on the growth of IMR-32 cells, we next studied the effect of indomethacin and NDGA on the growth inhibitory actions of AA and bleomycin. For this study, we used AA 30 µg/ml and bleomycin 60 µg/ml with or without indomethacin (20, 40, 60 µg/ml) or NDGA (5, 10, 20 µg/ml). AA, bleomycin and AA+bleomycin induced a significant inhibition in the growth of IMR-32 cells. At all the 3 concentrations tested, both indomethacin and NDGA produced a significant increase in the growth of IMR-32 cells (Fig. 16).

It may be noted here that the studies reported here were performed in two stages. The first set of experiments whose results were shown in Fig. 1 was performed with one lot of IMR-32 cells and the results shown in Fig. 8 were performed with another lot of IMR-32 cells. But it needs to be mentioned here that both lots of cells were obtained from the same source. This may explain mild discrepancy noted in the cytotoxic action of bleomycin on IMR-32 cells. For instance, bleomycin treatment reduced the cell viability to ∼80% (Fig. 8) at the end of 24 hrs of incubation when treated with 60 µg/ml. However, a similar bleomycin treatment at the same dosage and incubation hours reduced cell viability to ∼60% instead of ∼80% (Fig. 1). In addition, in Figs. 10–13, the baseline of bleomycin treatment effect was around 60% instead of 80% as shown in Fig. 8. All the results obtained were reconfirmed by repeating the studies at least twice and each time in triplicate with suitable controls to ensure the reproducibility of the results. These variations in the results suggest that even when the cells used in the study are from the same original source, there could be differences in their response to the same cytotoxic agent(s). This could be due to differences in the number of cycles at which the studies have been performed after the cells were revived. Nevertheless, we noted that the cytotoxic action of bleomycin and various PUFAs on IMR-32 cells studied were consistent except for a change in the percentage of viable cells. Such variations in the response of tumor and probably, normal cells to various cytotoxic agents under similar conditions is not surprising. For instance, previously we noted that the cytotoxic action of various PUFAs on various cancer cells may vary depending on the season the studies were performed [1]. We observed that the cytotoxic action of PUFAs on tumor cells were more evident during the winter compared to the results seen in the summer season. In other words, more number (percentage) of tumor cells were killed at the same concentration of fatty acid used when the studies were performed in winter compared to the cell killing noted in summer. This was a consistent finding when the experiments were done on two consecutive years. Thus, it is likely that season and cell cycle number at which the studies were performed could have a modulatory influence on the response of tumor cells to the cytotoxic agents that is in addition to the influence of the circadian rhythm on such responses.

#### Effect of AA and bleomycin on anti-oxidants, nitric oxide and lipid peroxides in IMR-32 cells *in vitro*

Bleomycin is known to enhance free radical generation and lipid peroxidation in cells [56]–[58], that may account for its radiomimetic actions. On the other hand, AA, being an unsaturated fatty acid, is known to be more susceptible for peroxidation [2], [59]. Hence, we studied the effect of bleomycin and AA alone or in combination on the amount of lipid peroxides formed and changes in anti-oxidants in IMR-32 cells *in vitro*. The results of these studies given in Tables 1 and 2, revealed that bleomycin and a combination of bleomycin+AA produced a significant increase in lipid peroxides in the supernatant (AA also increased lipid peroxides in the supernatant but was not statistically significant), while bleomycin alone and bleomycin+AA enhanced catalase, GST and GPX content, whereas bleomycin+AA enhanced only the content of SOD. There were no changes in the nitric oxide levels in the presence of bleomycin, AA and bleomycin+AA.

**Table 1:** Estimation of nitric oxide and lipid peroxidation in spent media and cell lysates of IMR-32 cells.

Group	Nitric Oxide (µM)		Lipid Peroxide (µM)	
	Supernatant	Lysate	Supernatant	Lysate
Control	0.74±0.01	0.73±0.01	0.57±0.05	0.44±0.012
BLM	0.74±0.01	0.72±0.02	1.02±0.16*	0.13±0.007*
AA	0.76±0.01	0.7±0.01	0.69±0.07	0.13±0.001*
AA+BLM	0.76±0.01	0.69±0.002	1.36±0.09*	0.11±0.004

IMR-32 cells were treated with AA 30 µg/ml and bleomycin 60 µg/ml and incubated for 24 hrs. At the end of treatment period, supernatant was collected and cells were lysed. All values are expressed as mean ± standard error (n = 3). *p<0.05 when compared to control. BLM = Bleomycin, AA = Arachidonic Acid.

**Table 2:** Estimation of anti-oxidants in cell lysates of IMR-32 cells.

Group	SOD (Units/mg protein)	Catalase (µM H_2_O_2_/min/gm protein)	GST (µM/min/gm protein	GPX (µM/min/gm protein
Control	9.639±1.42	298.85±74.71	13.95±1.19	532.37±34.8
BLM	9.792±3.46	1185.04±27.76*	33.29±1.67*	719.07±29.18*
AA	11.53±3.45	274.84±52.95	14.38±3.45	732.91±89.15
AA+BLM	20.732±4.28	387.05±6.34**	26.50±6.65	1112.67±64.7**

IMR-32 cells were treated with AA 30 µg/ml and bleomycin 60 µg/ml and incubated for 24 hrs. At the end of treatment period, supernatant was collected and cells were lysed. All values are expressed as mean ± standard error (n = 3). *p<0.05 when compared to control. **P<0.01 when compared to Bleomycin. BLM = Bleomycin, AA = Arachidonic Acid, SOD = Superoxide dismutase, GST = Glutathione-S-transferase, GPX = Glutathione peroxidase.

## Discussion

Both n-6 and n-3 PUFAs form precursors to many products that have several actions that also play a significant role in many diseases [60]–[62]. In this context, several studies have been performed to understand the role of PUFAs and their products in cancer, which have yielded controversial results [1]–[15], [23]–[42] especially with regard to the effect of prostaglandins, leukotrienes and thromboxanes on the proliferation of tumor cells. For instance, it was reported that some prostaglandins enhance the growth of tumor cells while other studies reported the opposite [27]–[39]. These diametrically opposite results could be due to the type of prostaglandins studied, the type of tumor cell under investigation, the dose of the compound and duration of exposure. Similarly, there is some controversy about the effect of various PUFAs on tumor cell proliferation. Some studies suggested that AA enhances the growth of tumor cells, while others reported an inhibitory action [27], [28]. It is noteworthy that n-6 LA has been reported to enhance the growth breast and lung cancer cells [63]–[66] while its derivative GLA was found to inhibit the growth [1], [2], [4], [26], [67]–[70]. These results suggest that a very minor change in the structure of the unsaturated fatty acid (LA has 18 carbon and 2 double bonds while GLA has 18 carbon and three double bonds) could have dramatic results in terms of their action on the growth of tumor cells. Furthermore, in none of these studies wherein various PUFAs were found to have either growth promoting or inhibitory action on tumor cells, neither their long-chain metabolites nor various products such as prostaglandins, leukotrienes and thromboxanes formed for these fatty acids were tested for their action on the growth of tumor cells under similar cell culture conditions. For instance, Rose et al [63]–[65] tested the effect of different doses of LA on the growth of breast cancer cells *in vivo* but never studied under the same conditions the effect of other fatty acids such as GLA, DGLA, AA, ALA, EPA and DHA and prostaglandins, leukotrienes and thromboxanes. In addition, in majority of these studies the effect of COX and LOX inhibitors were tested on the growth of tumor cells as a measure of the effect of eicosanoids formed from these fatty acids but never verified whether the results obtained with these inhibitors are in tune with the actions of their products such as prostaglandins, leukotrienes, thromboxanes.

In view of this, in the present we studied the effect of various PUFAs and their products including lipoxins, resolvins and protectins on the proliferation of IMR-32 cells *in vitro*. It is evident from the results obtained as shown in Fig. 2, all PUFAs were able to inhibit the growth of IMR-32 cells (EPA> DHA> ALA = AA = GLA> DGLA> LA). Of all the fatty acids tested, LA was the least effective in inhibiting the growth of IMR-32 cells (Fig. 2). In addition, LXA4, resolvin D1 and resolvin D2, 19,20 DiHDPA, 10(S),17(S) DiHDoHE, LTD4, LTE4, PGE1, PGE2 but not PGF2α, PGI2 showed significant inhibitory action on the growth of IMR-32 cells (Figs. 3–7) at the doses tested. LXA4, resolvin D1, resolvin D2, 19,20 DiHDPA, 10(S),17(S) DiHDoHE showed comparable growth inhibitory action on IMR-32 cells when tested at the same doses of 1, 5, and 10 ng/ml. In contrast to this, even at 10, 50 and 100 ng/ml doses, PGE1, PGE2, and LTD4 and LTE4 were much less effective in inhibiting the growth of IMR-32 cells (Figs. 6 and 7).

To our surprise, it was noted that indomethacin (20, 40 and 60 µg/ml), a COX inhibitor and NDGA (5, 10 and 20 µg/ml), a LOX inhibitor, enhanced the growth of IMR-32 cells at the highest doses (indomethacin at 60 µg/ml and NDGA at 20 µg/ml) tested, while at lower doses did not show any effect. If these results are any indication of the effect of COX and LOX products on the growth of IMR-32 cells, one would expect a significant decrease in the proliferation of IMR-32 cells in the presence of prostaglandins and leukotrienes. Though PGE1 and PGE2 and LTD4 and LTE4 did inhibit the growth of IMR-32 cells, their effect appeared much less dramatic compared to that of the highest doses of indomethacin and NDGA tested. For instance, PGE1 and PGE2 (at the highest dose of 100 ng/ml tested) inhibited the growth of IMR-32 cells ∼20% compared to the control whereas both PGF2α and PGI2 were without any inhibitory action (Fig. 6), while both LTD4 and LTE4 at the highest doses tested did not inhibit the growth ∼15% (Fig. 7). On the other hand, indomethacin and NDGA at the highest dose of 60 µg/ml and 20 µg/ml respectively tested, enhanced growth of IMR-32 cells to 180% of the control (Fig. 14), whereas at lower doses were without any effect. These results indicate that the growth enhancing actions of indomethacin and NDGA are unlikely to be due to the inhibition of formation of prostaglandins and leukotrienes. It is likely that this growth enhancing action of indomethacin and NDGA could be due to their non-specific anti-oxidant actions [71], [72].

In addition, in the present study we also evaluated the modulatory influence of various PUFAs and their products on the growth inhibitory actions of anti-cancer drug bleomycin on IMR-32 cells *in vitro*. Bleomycin was found to be a potent inhibitor of the growth of IMR-32 cells as shown in Fig. 1. Bleomycin, a glycopeptide antibiotic, that is used in the treatment of Hodgkin’s lymphoma as a component of ABVD (adriamycin, bleomycin, vinblastine and dacarbazine) and BEACOPP (bleomycin, etoposide, adriamycin, cyclophosphamide, oncovin = vincristine, procarbazine, prednisone), squamous cell carcinomas, and testicular cancer. The fact that PUFAs augment the cytotoxic action of bleomycin is in agreement of similar results reported previously that several unsaturated fatty acids have the ability to enhance the tumoricidal action of anti-cancer drugs [7], [18]–[23]. This synergism in the action of anti-cancer drugs and PUFAs on cancer cells could be attributed to their ability to enhance lipid peroxidation process and alter the status of anti-oxidants (Tables 1 and 2), though it has also been attributed to changes in the formation/concentrations of eicosanoids, PPARs, protein kinase C/extracellular signal regulated kinase pathway-dependent induction of c-Myc expression, Bbl-2 expression and Gs-axin-beta-catenin signaling axis in tumor cells [24], [25], [27]–[41]. This suggests that different tumor cells respond to PUFAs in their own specific manner, implying that no single mechanism could explain the proliferative and/or anti-proliferative actions of various PUFAs and their products. It is likely that the same type of tumor cells may show different responses to PUFAs and their products depending on their genetic heterogeneity, doses of PUFAs and eicosanoids used and the duration of exposure. Since PUFAs and their metabolites do possess pro- or anti-inflammatory actions and modulate immune response this could be yet another factor that needs to be taken in to consideration while studying their effects on tumor cells especially in an *in vivo* situation. The results of the present study showed that none of the prostaglandins, leukotrienes, lipoxins, resolvins and protectins tested enhanced the anti-cancer action of bleomycin *in vitro* except fatty acids. This indicates that when optimal dose(s) of PUFAs are used, tumor cells undergo apoptosis and also have the ability to augment tumoricidal action of anti-cancer drugs. The surprising observation that both indomethacin and NDGA not only abrogated the tumoricidal action of bleomycin but, in fact, enhanced the proliferation of IMR-32 suggesting that caution need to be exercised in their use (especially COX inhibitors) in the prevention of colon carcinoma where the use of COX-2 inhibitors have been recommended. Obviously, further studies are needed to understand how to tailor the dose and type of PUFAs in order to exploit their anti-tumoral action especially in an *in vivo* situation.

One pertinent question that crops up while extrapolating *in vitro* results reported here to an *in vivo* situation is how relevant are the doses of the PUFAs tested. It is known that the plasma AA concentration could be ∼5 µg/ml and sometimes reached values ten times higher than this maximal normal value in old patients who suffered from hypertension, in patients before and after various surgical procedures, in patients submitted to cardiopulmonary bypass with extracorporeal circulation or to abdominal aortic prosthesis, and in young volunteers or patients submitted to a strain test on bicycle ergometer [73]. On the other hand, that of LA can be ∼19.7±2.2; DGLA is ∼4.5±0.9; AA is ∼10.7±1.6; EPA ∼0.67±0.29; and DHA ∼3.6±0.6 g/100 g of total phospholipid from plasma of normal subjects [74], [75] suggesting that the concentrations of PUFAs tested in the present study are well within the physiological levels seen in the human plasma.
